# Dynamic Resource Optimization for Energy-Efficient 6G-IoT Ecosystems

**DOI:** 10.3390/s23104711

**Published:** 2023-05-12

**Authors:** James Adu Ansere, Mohsin Kamal, Izaz Ahmad Khan, Muhammad Naveed Aman

**Affiliations:** 1 Department of Electrical and Electronics Engineering, Sunyani Technical University, Sunyani P.O. Box 206, Ghana; jaansere@stu.edu.gh; 2 Electrical Engineering Department, National University of Computer and Emerging Sciences, Lahore 54770, Pakistan; 3 Department of Computer Science, Bacha Khan University, Charsadda 24420, Pakistan; azaz@bkuc.edu.pk; 4 School of Computing, University of Nebraska-Lincoln, Lincoln, NE 68588, USA

**Keywords:** robust joint resource optimization, energy efficiency, Lagrangian decomposition, Internet of Things, Kuhn–Munkres algorithm

## Abstract

The problem of energy optimization for Internet of Things (IoT) devices is crucial for two reasons. Firstly, IoT devices powered by renewable energy sources have limited energy resources. Secondly, the aggregate energy requirement for these small and low-powered devices is translated into significant energy consumption. Existing works show that a significant portion of an IoT device’s energy is consumed by the radio sub-system. With the emerging sixth generation (6G), energy efficiency is a major design criterion for significantly increasing the IoT network’s performance. To solve this issue, this paper focuses on maximizing the energy efficiency of the radio sub-system. In wireless communications, the channel plays a major role in determining energy requirements. Therefore, a mixed-integer nonlinear programming problem is formulated to jointly optimize power allocation, sub-channel allocation, user selection, and the activated remote radio units (RRUs) in a combinatorial approach according to the channel conditions. Although it is an NP-hard problem, the optimization problem is solved through fractional programming properties, converting it into an equivalent tractable and parametric form. The resulting problem is then solved optimally by using the Lagrangian decomposition method and an improved Kuhn–Munkres algorithm. The results show that the proposed technique significantly improves the energy efficiency of IoT systems as compared to the state-of-the-art work.

## 1. Introduction

The increasing growth in interconnected devices, i.e., the Internet of Things (IoT), has set the stage for new information and communication technologies (ICT) developments [1]. Recently, multiple practical IoT applications and businesses have proliferated worldwide [2]. In 2019, the Ericsson Mobility Report projected a growth of 7.4 billion smartphone subscriptions and 8.9 billion mobile communication broadband connections by the end of 2025 [3]. ICT infrastructure consumes about 3% of the energy resources and produces approximately 2% of the carbon dioxide emissions globally. The ICT industry is considered one of the key contributors to environmental footprint [4,5]. Besides the environmental concerns, telecommunication network operators are subject to financial pressure associated with energy consumption, as these costs can significantly reduce the total revenue in operational and capital expenditures [6,7]. With the emergence of 6G, its integration with the IoT network will provide large-dimensional unlimited connectivity, ultra-low transmission latency, and ultra-broad bandwidth [8]. Energy efficiency is a crucial consideration in the development of 6G-IoT networks, as these ubiquitous IoT applications and services are expected to connect billions of devices, and consume massive amounts of energy [9,10]. In consequence, having energy-efficient IoT applications will have a tangible influence on the environment, and will help in achieving long-term profitability for the network operators [11].

Conventional IoT devices are energy resource-constrained and battery-powered systems [12,13]. However, battery replacement and charging in some scenarios, where the IoT devices are deployed in remote or hostile environments, are costly and difficult to implement. Energy harvesting techniques [14] have been envisaged as promising solutions to provide constant energy to large-scale IoT networks. Energy harvesting systems obtain energy from hybrid sources—including wind, hydro, and solar energy—to enable autonomous power supply [15]. However, due to the uncertainties of time-varying environments, these methods cannot guarantee uninterrupted communication and continuous power supply to all the nodes in an IoT network. Currently, developing a novel energy resource allocation that optimizes energy efficiency performance for IoT systems is gathering increasing interest [16,17]. In [18], an efficient architectural design was proposed for IoT networks, enhancing the energy utilization efficiency of both its middleware and its hardware components [18]. He et al. [19] assumed perfect channel state information (CSI) knowledge for system throughput maximization in MIMO systems while satisfying quality of service (QoS) constraints. Due to channel quantization errors, inaccurate feedback, and CSI estimation errors, it is challenging to achieve effective CSI practically. Therefore, it is highly relevant to consider channel uncertainty conditions in designing an efficient resource allocation algorithm. Thus, imperfect CSI feasibility in MIMO systems has been examined for varying scenarios [20].

Further, Fang et al. [21] proposed a joint subchannel allocation and power allocation algorithm to enhance system performance and energy efficiency. Wang et al. [22] investigated multi-cell heterogeneous networks to jointly optimize power allocation, user association, and subchannel allocation in order to raise the weighted sum rate based on the Lagrangian decomposition method and bipartite theory to achieve optimal solutions globally. However, the authors neglected the optimization of the antennas at the BS, which is crucial in energy consumption. Dynamic resource allocation techniques are employed to improve the energy efficiency of IoT devices by dynamically adjusting the network topology, routing, and power levels based on the IoT device’s energy constraints and communication requirements [23,24]. Most of the above existing methods are mixed non-linear optimization problems, which are NP-hard due to the huge and complex nature of IoT systems with continuous and discrete decision variables. IoT systems involve numerous interconnected devices that generate a large multi-dimensional data, and these devices may have different characteristics such as varying power allocation, battery storage, and energy constraints. This makes the non-convex optimization of dynamic large-scale IoT systems a challenging task. By employing this strategy, 6G-IoT networks can achieve high levels of energy efficiency, reduce their environmental impact, and enable the deployment of sustainable IoT solutions [25]. Despite the potential benefits of energy efficiency in 6G-IoT networks, there are several challenges, such as network complexity and limited energy resources, that need to be addressed to satisfy realistic implementation and successful deployment in dynamic, large-scale IoT environments.

This paper explores dynamic resource allocation for joint optimization of the number of activated RRUs, subchannel allocation, user selection, and power allocation to enhance system performance and energy efficiency in dynamic large-scale IoT systems, subject to the transmit power and QoS requirements for all IoT devices. This paper was inspired by the aforementioned studies. Due to the non-convexity, the formulated problem is intractable and NP-hard, which means there is no effective method to obtain the optimal solution in polynomial time. By exploiting fractional programming properties, we design a framework, i.e., a dynamic resource algorithm, using Lagrangian decomposition and the Kuhn–Munkres (KM) algorithm in order to optimally solve it. In this paper, we summarize the technical contributions as follows:

1. We design an energy-efficient resource allocation framework and formulate a non-convex MINLP problem for joint optimization of user selection, subchannel allocation, user selection, power allocation, and the number of activated RRUs in order to enhance the energy efficiency in dynamic large-scale 6G-IoT ecosystems.
2. In order to decompose the problems of non-convex optimization into small segments, we leverage the fractional programming property. We propose the Lagrangian decomposition method to optimize power allocation and the KM algorithm to dynamically allocate resources to IoT users to obtain optimal solutions. This can significantly reduce the computational complexity and make the optimization process more scalable in dynamic large-scale IoT systems.
3. The effectiveness of the proposed algorithm compared to the leading-edge approaches in the form of energy efficiency gain is verified through extensive simulations.

The rest of the paper is organized as follows: Section 2 describes the architectural design of the considered system model. Section 3 investigates resource allocation and formulates an optimization problem whilst Section 4 presents the proposed optimal joint resource allocation algorithm. Section 5 examines the performance evaluations and discussions. Finally, the study is concluded in Section 6.

## 2. IoT Network Model

**Figure 1:**
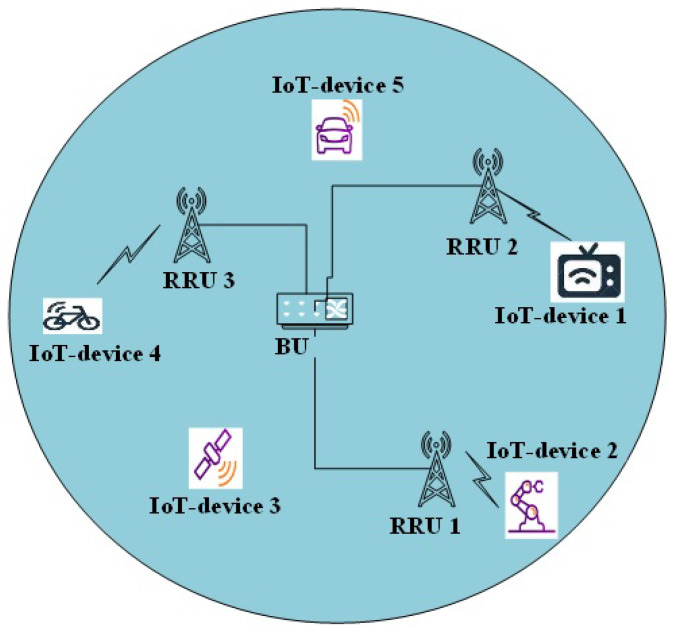
Illustration of downlink IoT systems.

We examine a downlink IoT system, consisting of a baseband units (BU) pool, which connects the *N* remote radio units (RRUs), as illustrated in Figure 1. Each RRU is well-equipped and has a single antenna to serve the *K* IoT device for transmitting and receiving radio frequency signals. The system resources are allocated to the IoT devices orthogonally to avoid inter-IoT devices’ interference. The maximum number of large-scale antennas, $L_{\max}$, is at the RRU, where the activation of RRUs is performed to increase effective communication among the IoT devices. The resource allocation in IoT systems improves power allocation to different IoT devices based on channel conditions, as more power is allocated to IoT devices with weaker channel conditions. With perfect CSI at the transmitter, RRU stores energy temporarily in transmitting data to the neighbor IoT device. Moreover, the IoT device senses the subchannel in an opportunistic mode through RRU, while the IoT device is assigned to one RRU. We assume the RRUs operate as a relay protocol to forward the received signals from the IoT device to the centralized baseband unit (BU). The uncertainties in the communication channels are independent and identically distributed (Gaussian) to obey the Rayleigh fading requirements.

### 2.1. Channel Model and Estimation

Considering the downlink training phase, it is assumed that all the IoT devices simultaneously transmit pilot succession for channel estimation at τ≥K, where τ is the size of each pilot succession. A set of pilot succession is orthogonally related to $\Phi =\left[\phi_{1},\phi_{2},\ldots ,\phi_{K}\right]\in C^{\tau \times K}$, which are arbitrarily allotted to the IoT devices and satisfy $\Phi^{H}\Phi =I_{K}$

Assuming that all the antennas in this phase are activated, the received signal at the *n*-th RRU is given by

(1) $$Y_{k}=\sum_{k=1}^{K}\sqrt{p_{k}}H_{k}^{T}\Phi +Z_{k}^{T}$$

where $p_{k}$ denotes the transmit power of *k*-th IoT device, $H_{n}=\left[h_{n,1},h_{n,2},\ldots ,h_{n,K}\right]\in C^{L_{\max}\times K}$ is the channel matrix from the *n*-th RRU to the *k*-th IoT device, and $Z_{k}^{T}$ is complex Gaussian noise, for which the distribution is $\mathrm{CN}(0,\sigma_{k}^{2})$. The communication channel is modeled as $h_{n,k}=\sqrt{\alpha_{n,k}}g_{n,k}$, and represents the channel vector for the *n*-th RRU and *k*-th IoT devices. In addition, both $\alpha_{n,k}$ and $g_{n,k}\in R^{N\times 1}$ denote the coefficient of large-scale and small-scale fading channels between *n*-th RRU and *k*-th IoT devices, respectively.

By accessing the channel estimation gain, it is assumed that the ${\tilde{y}}_{n,k}$ is projected onto $\Phi_{k}$ as

(2) $${\tilde{y}}_{n,k}^{T}\overset{\Delta }{\;=}Y_{n}^{T}\Phi^{H}=\tau \sum_{k=1}^{K}\sqrt{p_{k}}h_{n,k}^{T}+{\tilde{Z}}_{k}^{T}$$

Applying the minimum mean square error (MMSE) channel estimator method [26], the channel estimated, $h_{n,k}$, from *n*-th RRU to *k*-th IoT devices is given as

$${\tilde{h}}_{n,k}=\frac{E\left\{h_{n,k}{\tilde{y}}_{n,k}^{*}\right\}}{E\left\{{\left|{\tilde{y}}_{n,k}^{*}\right|}^{2}\right\}}{\tilde{y}}_{n,k}^{*}$$

(3) $$=\frac{\sqrt{p_{k}}\alpha_{n,k}}{\tau p_{k}\alpha_{n,k}+\sigma_{k}^{2}}\left(\tau \sqrt{p_{k}}h_{n,k}+\phi_{k}Z_{k}^{T}\right)$$

Hence, the channel estimation error, $\epsilon_{n,k}$ is stated as $\epsilon_{n,k}=h_{n,k}-{\hat{h}}_{n,k}$, having a distribution of $\epsilon_{n,k}∼\mathrm{CN}\left(0,\alpha_{n,k}I_{K}\right)$.

### 2.2. Data Transmission Model

It can be assumed that each deployed RRU transmits data information to the connected IoT devices. $g_{n,k}\in R{}^{N\times 1}$ and $h_{n,k}\in R{}^{N\times 1}$ represent the RRU and channel vector from *n*-th RRU to *k*-th IoT devices, respectively. However, the $x_{k}$ represents the transmitted signal to the *k*-th IoT device and can be expressed as *k*-th IoT devices, which is given by

(4) $$x_{n,k}=\sum_{k=1}^{K}\sqrt{p_{n,k}}{\hat{h}}_{n,k}g_{n,k}$$

Therefore, the received signal of *k*-th IoT device on subchannel $i\in \{1,2,3,\ldots K_{n}\}$ is given by

$$y_{n,k}=\sum_{n=1}^{N}\sqrt{p_{n,k}}h_{n,k}^{T}x_{n,k}+z_{n,k}$$

(5) $$=\sum_{n=1}^{N}\sqrt{p_{n,k}}h_{n,k}^{T}{\tilde{g}}_{n,k}x_{k}+\sum_{n=1}^{N}\sum_{l=1,l\neq k}^{K_{n}}\sqrt{p_{n,l}s_{n,l}}h_{n,l}^{T}{\tilde{g}}_{n,l}x_{l}+z_{n,k}$$

where $z_{n,k}$ denotes Gaussian noise with zero mean and unit variance, and $s_{n,l}$ represents the subchannel.

The achievable rate for the *n*-th RRU to *k*-th IoT devices is

(6) $$r_{n,k}=B\log_{2}\left(1+\gamma_{n,k}\right)$$

where *B* represents the bandwidth and $\gamma_{n,k}$ is the signal-to-interference-plus-noise ratio (SINR) [27]. The $\gamma_{n,k}$ is given as

(7) $$\gamma_{n,k}=\frac{p_{n,k}{\left|h_{n,k}^{T}{\tilde{g}}_{n,k}\right|}^{2}}{\sum_{l=1,l\neq k}^{K_{n}}\sum_{n=1}^{N}{\left|h_{n,k}^{T}{\tilde{g}}_{n,k}\right|}^{2}p_{n,l}s_{n,l}+\sigma_{n,k}^{2}}$$

Therefore, the maximum achievable rate, $R_{n,k}$, of *n*-th RRU to *k*-th IoT devices is expressed as

(8) $$R_{n,k}=\sum_{n=1}^{N}r_{n,k}.$$

### 2.3. The Power Consumption Model

The power consumption at the RRUs and power amplifiers forms the largest portion of the entire power consumption in the downlink system [28]. The sum power consumption comprises the RF transmit power, the fixed power consumption $P_{\mathrm{FIX}}$ for site cooling and load processing, and the circuit power consumption $P_{c}$ from the activated RRUs. As a result, the total power consumption is modeled by

$$P_{T}=P_{\mathrm{FIX}}+P_{t}+P_{c}$$

(9) $$P_{T}=P_{\mathrm{FIX}}+\sum_{n=1}^{N}\sum_{k=1}^{K}\frac{1}{\eta_{e}}p_{n,k}+p_{s}\sum_{n=1}^{N}L,$$

where $P_{c}=p_{s}\sum_{n=1}^{N}L$ represents the circuit power consumption, $p_{s}$ is the power cost for serving the deployed RRUs, and L characterizes the large-scale deployed RRUs. $P_{t}=\sum_{k=1}^{K}\sum_{n=1}^{N}\frac{1}{\eta_{e}}p_{n,k}$ is the transmit power, and $\eta_{e}$ indicates the power amplifier efficiency, $\eta_{e}\in \left\{0,1\right\}$.

## 3. Resource Allocation and Optimization Problem

This section investigates the resource allocation problem in order to formulate an optimization task towards maximizing energy efficiency performance.

### 3.1. Energy Efficiency Optimization

Energy efficiency, η, is defined as the achievable rate $R_{n,k}$ to the overall power consumption $P_{T}$ of the considered system (bits/Joule) [29]. Thus, energy efficiency η can be expressed in terms of power allocation P, activated RRUs selection A, user selection U, and subchannel allocation S:

(10) $$\eta \left(P,A,U,S\right)=\frac{R_{n,k}\left(P,A,U,S\right)}{P_{T}\left(P,A,U,S\right)}$$

### 3.2. Formulation of Optimization Problem

The joint optimization of power allocation P, activated RRUs selection A, user selection U, and subchannel allocation S is now described. Mathematically, the formulated optimization problem of the considered system is

(11) $$\begin{array}{c} P1:\max_{P,A,U,S}\eta \left(P,A,U,S\right)\\ C1:\sum_{n=1}^{N}\sum_{k=1}^{K}s_{n,l}p_{n,k}\leq \eta_{e}P_{\max},\forall k,\forall n\\ C2:\sum_{n=1}^{N}\sum_{k=1}^{K}s_{n,l}R_{n,k}\geq R_{\min},\forall k,\forall n\\ C3:\sum_{n=1}^{N}p_{n,k}\leq \delta_{o},n\in \psi \\ \begin{array}{c} C4:\sum_{n=1}^{N}s_{n,l}=1,s_{n,l}\in \left\{0,1\right\},\forall n,l\\ C5:\sum_{n=1}^{N}u_{n,k}=1,u_{n,k}\in \left\{0,1\right\},\forall n,k \end{array}\\ C6:p_{n,k}\geq 0,\forall k,\forall n\\ C7:0\leq L\leq L_{\max},L_{\max}\in Z^{+} \end{array}$$

where $Z^{+}$ denotes the set of positive integers and ψ is the feasible region. The optimization constraints in P1 are defined as follows: C1 denotes the transmit power constraint boundary for the RRU and $P_{\max}$ represents the maximum transmit power. C2 guarantees the QoS requirements for all IoT devices and $R_{\min}$ is the minimum data rate required. C3 ensures that $p_{n,k}$ restricts inter-user interference and $\delta_{o}$ is the predefined threshold. C4 and C5 guarantee that at most one IoT device is selected for one RRU,. C6 represents the power allocation boundary and C7 indicates the combinatorial constraint on the deployed RRUs. However, the objective function in P1 is a mixed non-linear optimization problem, which is NP-hard, with constraints involving non-linear functions making it difficult to solve for the optimal solution. The P1 involves a combinatorial optimization over the multi-dimensional discrete decision variables. Moreover, tackling the P1 in polynomial time becomes exponentially harder as the optimization problem size becomes larger. Hence, obtaining the optimal solution in real-time scenarios is computationally costly in dynamic large-scale IoT environments. Therefore, we transform the considered system problem into a convex form and design a novel dynamic resource allocation technique to optimally solve it.

### 3.3. Novel Dynamic Resource Allocation Design

Due to the intractability of P1, we develop a novel algorithm for iterative resource allocation in order to solve the transformed problem as discussed in the following subsections.

#### Transformation of Energy Efficiency Optimization

The P1 is a mixed-integer non-linear programming (MINLP) problem, which is a non-convex problem and is NP-hard, with no practical solutions in polynomial time. To address these challenges, we transform the energy efficiency maximization problem into convex form. By exploiting fractional programming, we can convert P1 into a convex problem in parametric form. Let Ω represent the feasible solutions of $\left(P,A,U,S\right)$. The optimal energy efficiency $\eta^{*}$ can be expressed as

(12) $$\eta^{*}\left(P^{*},A^{*},U^{*},S^{*}\right)=\max_{\left(P,A,U,S\right)\in \Omega }\frac{R_{n,k}\left(P,A,U,S\right)}{P_{T}\left(P,A,U,S\right)}$$

where $\left(P^{*},A^{*},U^{*},S^{*}\right)$ denote the optimal solutions. Therefore, the P1 is transformed into a parametric form as

(13) $$\begin{array}{c} P2:\max_{\left(P,A,U,S\right)\in \Omega }\left[R_{n,k}\left(P,A,U,S\right)-\eta^{*}P_{T}\left(P,A,U,S\right)\right]\\ s.t.:C1-C7 \end{array}$$

We provide the following theorem to enable the transformation procedure in P1.

**Theorem** **1.** *Energy Efficiency*

*If and only if*

(14) $$\begin{array}{c} \max_{\left(P,A,U,S\right)\in \Omega }\left[R_{n,k}\left(P^{*},A^{*},U^{*},S^{*}\right)-\eta^{*}P_{T}\left(P,A,U,S\right)\right]\\ =R_{n,k}\left(P^{*},A^{*},U^{*},S^{*}\right)-\eta^{*}P_{T}\left(P,A,U,S\right)=0 \end{array}$$

*for $R_{n,k}\left(P,A,U,S\right)\geq 0$ and $P_{T}\left(P,A,U,S\right)\geq 0$.*

**Proof.**  The proof of Theorem 1 follows a similar method as that presented in [30]. Thus, Theorem 1 illustrates an objective function in the equivalent subtractive form as $R_{n,k}\left(P,A,U,S\right)-\eta^{*}P_{T}\left(P,A,U,S\right)$. To tackle the resource-constrained problem, we first solve (12) iteratively for the current η value, and update it until it reaches $\eta^{*}\geq 0$. However, P2 is still MINLP and very difficult to solve.    □

## 4. Proposed Joint Optimal Iterative Method

In this section, the proposed optimization problem is relaxed and the time-sharing requirements to assign a subchannel for each IoT device are applied.

### 4.1. Relaxed Problem Formulation

The problem P2 is a convex function and exhibits fractional programming properties, and it is maximized over a convex set. To solve the MINLP in P2 and for further decomposition, it is important to relax the optimization problem. In addition, the designed resource allocation algorithm decomposes the objective function to mitigate inter-user interference. Therefore, the binary variables $s_{n,l}\in \left\{0,1\right\}$ and L from the objective function in P1 are relaxed to continuous variables as follows.

(15) $$\sum_{n=1}^{N}s_{n,l}=1,s_{n,l}\in \left\{0,1\right\},\forall n$$

(16) $$0\leq L\leq L_{\max},L_{\max}\in Z^{+}$$

Each IoT device will possibly interfere with the neighbor IoT devices using the same subchannel. In real-world applications, the multiuser interference limit is introduced to avoid multiuser interference among IoT devices. Moreover, we relax $s_{n,l}$ in C4 and C7 to be a real variable, as $\left[0,1\right]$, to indicate that the subchannel is allocated. Through continuous relaxations, the constraints C4 and C7 in (11) remain a non-convex pairing constraint and can be tackled using the Lagrangian dual decomposition method. Hence, $s_{n,l}$ is defined as a time-sharing condition for *n* IoT devices to transmit data through subchannel *l*. Denoting time-shared activated RRUs as ${\tilde{L}}_{n,k}=s_{n,l}L_{n,k}$ and time-shared power as ${\tilde{p}}_{n,k}=s_{n,l}p_{n,k}$, the relaxed C4 and C7 for the P2 can be reformulated as

(17) $$\begin{array}{c} \begin{array}{c} P3:\max_{P,A,U,S}\eta \left(P,A,U,S\right)\\ C1:\sum_{n=1}^{N}\sum_{k=1}^{K}s_{n,l}p_{n,k}\leq \eta_{e}P_{\max},\forall k,\forall n\\ C2:\sum_{n=1}^{N}\sum_{k=1}^{K}s_{n,l}R_{n,k}\geq R_{\min},\forall k,\forall n\\ C3:\sum_{n=1}^{N}p_{n,k}\leq \delta_{o},n\in \psi \\ \begin{array}{c} C4:\sum_{n=1}^{N}s_{n,l}=1,s_{n,l}\in \left\{0,1\right\},\forall n,l\\ C5:\sum_{n=1}^{N}u_{n,k}=1,u_{n,k}\in \left\{0,1\right\},\forall n,k \end{array}\\ C6:p_{n,k}\geq 0,\forall k,\forall n\\ C7:0\leq L\leq L_{\max},L_{\max}\in Z^{+} \end{array}\\ C8:\sum_{l\neq k}\left(\frac{2}{\chi }\right)p_{n,l}s_{n,l}\leq \Phi ,\forall n,l \end{array}$$

where constraint, *C*8, represents the maximum inter-user interference and χ is the outage probability. The transformed problem P3 in (17) becomes a jointly optimized concave with respect to each optimization variable. The objective function in P3 exhibits non-convexity, and therefore the dual decomposition method is applied to address this optimization problem.

### 4.2. Dual Decomposition Problem

Next, we will describe the optimization problem for power allocation, the activated RRUs, and subchannel allocation by exploiting the Lagrangian decomposition method. The Lagrangian function is given by

(18) $$\begin{array}{c} L\left(\rho ,\lambda ,\mu ,P,A,U,S\right)=\sum_{k=1}^{K}\left(w_{k}+\lambda \right)\sum_{n=1}^{N}R_{n,k}-\lambda R_{\min}\\ +\rho \left(\eta_{e}P_{\max}-\sum_{n=1}^{N}\sum_{k=1}^{K}p_{n,k}\right)\\ -\eta \left(P_{\mathrm{FIX}}+\sum_{n=1}^{N}\sum_{k=1}^{K}\frac{1}{\eta_{e}}p_{n,k}+p_{s}\sum_{k=1}^{K}L\right)\\ -\sum_{k=1}^{K}\sum_{l=1}^{L}\mu_{l,k}\left(\sum_{j\neq k}\left(\frac{2}{\chi }\right)p_{n,l}s_{n,l}-\Phi \right) \end{array}$$

where $w_{k}$ represents the weight for *k*-th IoT device, ρ≥0, λ≥0 and μ≥0 are the Lagrange multipliers for power allocation constraint, minimum outage probability constraint, and Φ is the inter-IoT device interference constraint, respectively. It can be seen that the dual optimization problem is continually convex, which is given by

(19) $$\min_{\rho ,\lambda ,\mu >0}\max_{P,A,U,S}L\left(\rho ,\lambda ,\mu ,P,A,U,S\right)$$

Iteratively, the dual problem can be separated into two forms. Initially, the *inner loop* is considered to enhance power allocation, and subchannel allocation which is activated using the Lagrange multipliers. Secondly, the *outer loop*, a master dual problem that reduces the complexity from the Lagrange multipliers, is explored. In each iteration, the IoT device applies the local information to tackle the subproblems.

### 4.3. Inner Loop Method

A systematic approach is provided to reach a global optimal solution for the proposed problem by introducing a standard Lagrangian dual decomposition method. Using the set of Lagrange multipliers (ρ,λ,μ), the dual decomposition method [31] is reformulated as

(20) $$\max_{P,A,U,S}\left(\rho ,\lambda ,\mu ,P,A,U,S\right)$$

According to the Karush–Kuhn–Tucker (KKT) condition [31], the optimal solutions of the inner loop method are obtained as demonstrated in the subsequent sections.

#### 4.3.1. Optimal Power Allocation

By differentiating L(ρ,λ,μ,P,A,U,S) with respect to $p_{n,k}^{*}$ and rearranging them in terms of $p_{n,k}^{*}$ based on the KKT condition [31], the optimum power allocation $p_{n,k}^{*}$ of *k*-th IoT device on *n*-th subchannel is achieved for

(21) $$p_{n,k}^{*}=\left[\frac{\left(1-\chi \right)B\left(w_{k}+\lambda \right)}{\left(\rho +\eta \varphi +\sum_{j\neq k}\left(\frac{2}{\chi }\right)p_{n,l}s_{n,l}\right)\ln\left(2\right)}\right]\;$$

where φ≥1 is assumed to be constant and accountable to power consumption and *B* is the bandwidth. The power allocation displays a multiuser, water-filling procedure. The inter-IoT device’s interference is minimized when the power allocation is high to satisfy the constraint C7 and where η≥0 prevents power consumption.

#### 4.3.2. Optimal Number of Activated RRUs Allocation

Likewise, differentiating L(ρ,λ,μ,P,A,U,S) with respect to $L_{n,k}^{*}$ and reorganizing them in terms of $L_{n,k}^{*}$, the closed-form expression for the optimal number of activated RRUs based on KKT condition, according to *k*-th IoT device on *n*-th subchannel, is given by

(22) $$L_{n,k}^{*}={\left[\left\{\frac{\left(1-\chi \right)B\left(\max_{k\in \phi_{j}}w_{k}+\lambda \right)}{\ln\left(2\right)\left(\frac{\eta }{\alpha_{j}}\right)P_{c}}\right\}\right]}_{L}^{L_{\max}}$$

where $\phi_{j}$ represents a nominated IoT device set depending on subchannel *n*, and $\alpha_{j}$ accounts for the number of weighted IoT devices that have an equivalent maximum value allocated to each selected IoT device. At a severe data rate strategy of C2 in P3, the dual variable λ becomes adequately scalable to improve the resource allocation to distribute more RRUs to all scheduled IoT devices, as illustrated in (22), to satisfy constraint C2. Moreover, (22) shows that each IoT device will, in due course, operate with the equivalent number of activated RRUs.

#### 4.3.3. Optimal Subchannel Allocation

In addition, the optimal subchannel allocation is obtained by taking the derivative of the L(ρ,λ,μ,P,A,U,S) with respect to $L_{n,k}^{*}$. Therefore, the optimal subchannel allocation is given as

(23) $$\frac{\partial L(\rho ,\lambda ,\mu ,P,A,U,S)}{\partial s_{n,l}^{*}}=Z_{n,l}$$

where $Z_{n,l}\geq 0$ represents the differential cost for assigning *n* subchannel to *k*-th IoT device, which is expressed as

(24) $$\begin{array}{c} Z_{n,l}=\left(1-\chi \right)B\left(w_{k}+\lambda \right)\left(\log_{2}\left(\frac{p_{n,k}^{*}}{\Phi +B\sigma_{n,k}^{2}}\right)\right.\\ +\left.\log_{2}\left(L_{n,k}^{*}\left(1-\sigma_{n,k}^{2}\right)\left(1-\delta \right)\right)-2/\ln\left(2\right)\right) \end{array}$$

It is significant that the $Z_{n,l}\geq 0$ allows the IoT devices with a positive allocated data rate on the subchannel to select the positive minimal cost to enhance the system’s performance. Hence, the subchannel allocation *k* at the number of activated RRU is centered on the following criterion:

(25) $$s_{n,l}^{*}=\left\{\begin{array}{c} 1,Z_{n,l}\geq 0\\ 0,\mathrm{otherwise} \end{array}\right.$$

### 4.4. Outer Loop: Master Subproblem Solution

According to the optimal solutions of $p_{n,k}^{*}$, $L_{n,k}^{*}$ and $R_{n,k}^{*}$, an iterative method is required to solve the master problem. Since the dual optimization problem of (19) is not differentiable, a sub-gradient method can be applied in updating the dual variables as follows:

(26) $$\rho \left(\tau +1\right)={\left[\rho \left(\tau \right)-\beta_{1}\left(\tau \right)\times \left(P_{\max}-\sum_{n=1}^{N}\sum_{k=1}^{K}p_{n,k}\right)\right]}^{+}$$

(27) $$\lambda \left(\tau +1\right)={\left[\lambda \left(\tau \right)-\beta_{2}\left(\tau \right)\times \left(\sum_{n=1}^{N}\sum_{k=1}^{K}s_{n,l}R_{n,k}-R_{\min}\right)\right]}^{+}$$

(28) $$\mu \left(\tau +1\right)={\left[\mu \left(\tau \right)-\beta_{3}\left(\tau \right)\times \left(\Phi -\sum_{j\neq k}\left(\frac{2}{\chi }\right)p_{n,l}s_{n,l}\right)\right]}^{+}$$

where $\beta_{j}\left(\tau \right),\tau \in \left\{1,2,3\right\}$ denotes the positive step sizes and τ≥0 represents the iteration index. The Lagrange multipliers in (26)–(28) are updated based on the sub-gradient approach to maximize energy efficiency performance.

### 4.5. Optimal User Selection

This section aims to apply the Kuhn–Munkres (KM) algorithm to transform optimization problems into maximum-weight matching of the bipartite graph in a combinatorial manner. KM algorithm solves the optimal assignment problem with the substantial minimum cost in polynomial time in order to enhance the energy efficiency of large-scale IoT networks.

Let $G=\left(\Omega_{1},\Omega_{2},E\right)$ represent an undirected graph, where $\Omega_{1}$ and $\Omega_{2}$ are the vertices and *E* is the edge connectivity. However, the $\Omega_{1}$ and $\Omega_{2}$ specify RRUs and IoT devices. Every vertex of $\Omega_{1}$ is connected to that $\Omega_{2}$, and no edge connects to the same set of vertices. A perfect matching in $G=\left(\Omega_{1},\Omega_{2},E\right)$ is attained as a matching *M* that forms a set of M⊆E having pairwise non-adjacent edges. Letting $H_{\ell }$ signify the subgraph of *G* that involves the connected edges, the maximum-weight matching of an undirected graph *G* is determined as a matching *M* having the largest weight. Based on Equation (11), the formulated optimization problem for user selection is given by

(29) $$\begin{array}{c} P4:\max_{U}\eta \left(P,A,U,S\right)\\ s.t.:\\ C5:\sum_{n=1}^{N}u_{n,k}\leq 1,u_{n,k}\in \left\{0,1\right\},\forall n,k \end{array}$$

The P4 is an optimization problem with MINLP properties. The KM algorithm is introduced, and it depends on a complete bipartite graph with a feasible labeling *ℓ* vertex in order to find an optimal matching problem to obtain maximum-weight matching $w_{t}$ with reduced complexity. Thus, the shortest path searching technique is applied to achieve an optimal user selection solution. Each iteration finds an augmenting path with maximum-weight relative to the existing matching *M*.

However, all IoT devices in the network are uniformly distributed and can connect to only one RRU. This resource allocation binary index for *k*-th IoT devices connected to *n*-th RRU is defined as

(30) $$u_{n,k}=\left\{\begin{array}{c} 1,k-\mathrm{th}\;IoT\;\mathrm{connects}\;n-\mathrm{th}\;RRU,\\ 0,\mathrm{otherwise}. \end{array}\right.$$

Let $\Omega_{1}=\left\{RRU_{1},RRU_{2},...,RRU_{N}\right\}$ and $\Omega_{2}=\left\{IoT_{1},IoT_{2},...,IoT_{K}\right\}$ mean the set of vertices of RRUs and IoT devices, respectively. Let *X* and *Y* be matched connected edges. Therefore, the KM algorithm can be applied to attain optimal user selection in the maximum weight of connected edges in $\Omega_{1}$ and $\Omega_{2}$ from *n*-th RRU to *k*-th IoT device from the practical steps as follows:

1. Initialize perfect matching *M* and feasible labelling *ℓ* in ℓ(v).
2. Set $S=\left\{v\right\},T=\theta$.
3. If *ℜ* denotes an optimal matching of complete bipartite graph *G*, the Equation (29) can be optimally solved.
4. Otherwise, select vertex v∈X.
5. If $T=H_{\ell }\left(S\right)\neq Y$ and S⊆X, then set $H_{\ell }\left(S\right)=T$.
6. Update the feasible labels as
  (31) $$\beta_{\ell }=\min_{s\in S,y\notin T}\left\{\ell \left(x\right)+\ell \left(y\right)-w_{t}\left(x,y\right)\right\}$$
  where the new feasible label $\ell^{\prime }\left(v\right)$ can be expressed as
  (32) $$\ell^{\prime }\left(v\right)=\left\{\begin{array}{c} \ell \left(v\right)-\beta_{\ell }\;\mathrm{if}\;v\in S\\ \ell \left(v\right)+\beta_{\ell }\;\mathrm{if}\;v\in T\\ \ell (v),\;\;\mathrm{otherwise}. \end{array}\right.$$
  iff $\beta_{\ell }>0$ and $H_{\ell^{\prime }}\left(S\right)\neq T$.
7. If $H_{\ell }\left(S\right)\neq T$, set $y\in H_{\ell }\left(S\right)-T$, and go to step 2.

After the finite number of iterations, the termination condition occurs to guarantee the optimum solution. Therefore, the optimal user selection is achieved from the optimal matching solution of *G*.

### 4.6. Proposed Joint Resource Allocation Algorithm

The proposed joint energy-efficient resource allocation (JEERA) algorithm is investigated to jointly optimize power allocation, activated RRUs, subchannel allocation, and user selection allocation to enhance energy efficiency gain as implemented in Algorithm 1. The Lagrangian decomposition technique and KM algorithm are employed to achieve near-optimal solutions. It has been shown that the optimization problem in *P*1 is considered as a large-scale MINLP and has no practical solutions in polynomial time. From Algorithm 1, the ($P^{*},A^{*},U^{*},S^{*}$) and $\eta^{*}$ are sequentially optimized with guaranteed convergence. Furthermore, the optimality condition of Algorithm 1 is similar to the Dinkelbach method, where the linear objective function has updated parameters in each iteration, with reduced computational complexity.
**Algorithm 1:** Proposed JEERA Algorithm to Maximize Energy Efficiency
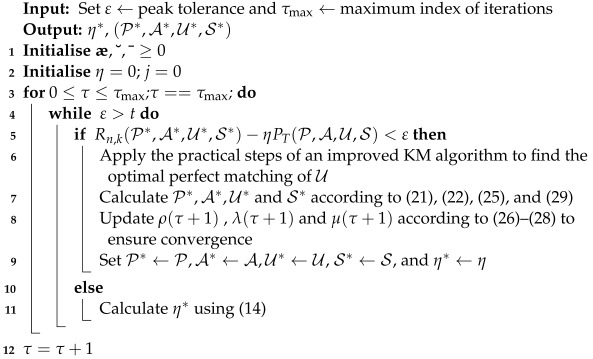


Algorithm 1 is implemented in a centralized mode through the control unit, having fixed system parameters such as maximum transmit power $P_{\max}$, and minimum rate requirement and number of RRUs to achieve optimal solutions. The control unit disseminates optimal solutions to all RRUs and iteratively updates $\rho \left(\tau +1\right)$, $\lambda \left(\tau +1\right)$ and $\mu \left(\tau +1\right)$, according to (26)–(28) at low computational complexity. The convergence of Algorithm 1 will be illustrated under performance evaluations in Section 5.1.5.

### 4.7. Computational Complexity and Feasibility

This section analyzes the complexity of the proposed JEERA algorithm for each iteration. Initially, each iteration is set to compute KL and allocate *l*-th subchannel to *k*-th IoT device. The *K* iterations are performed to assign only one RRU to each *K* IoT device. Hence, the complexity for the initial stage is $LK^{2}$. In the second stage, each iteration shows complexity for the sub-gradient approach, which is given by $O\left(KL\right)$, confirming convergence as $O\left(\left(K+1\right)\right)$ in a few iterations. However, the sub-gradient method has a complexity of $O\left(KL{\left(K+1\right)}^{2}\right)$. According to bisection searching [31], the total complexity obtained is OKL(K+1)2.log2(11θθ), where θ is the required accuracy to support the bisection search.

In the third stage, the subchannel allocation is fixed and therefore has computation O(L) only at each iteration. Thus, the complexity is assumed to be OL(K+1)2.log2(11θθ). As observed from the given analysis, the iteration in the initial stage is constrained by the *K* IoT device, since one RRU is allocated only to one IoT device. The complexity of the proposed JEERA algorithm used in the second and third iterations is efficiently evaluated by OL(K+1)3.log2(11θθ). This shows that the proposed algorithm has polynomial complexity and enables practical implementation.

## 5. Performance Evaluation and Discussion

In this section, we investigate the performance of the proposed algorithm through computer simulations and compare it with the baseline algorithms. A single cell with a radius of 1 km is considered and the IoT users are randomly and uniformly distributed between the reference distance of 40 m. The large-scale fading of the downlink channels and inter-IoT user channels is modeled as Rayleigh fading, similar to the 3GPP-Urban Micro model [32], which is independent and identically distributed, in accordance with the path-loss aa 128.1+40.2log10d where *d* denotes the distance (in km) between the IoT device users and RRU [20,33]. In the following, the simulation results are obtained by averaging the 10 instances of the IoT network. All simulation results are obtained by estimating an average of 1500 channel communications. Other simulation parameters are listed in Table 1.

Moreover, we compare the performance of the proposed JEERA algorithm with the existing algorithms in [27,30] as follows: the joint optimization for user association, subchannel allocation, and power allocation (JUSAP) algorithm to maximize the weighted spectral efficiency [27]; and a designed framework to jointly optimize the power allocation, user selection, and precoding (JPAUP) algorithm to maximize the weighted sum-rate [30]. However, for the JUSAP algorithm, the authors neglected to optimize the deployed RRUs, which is crucial in energy consumption. Additionally, the authors of the JPAUP algorithm overlooked optimizing the deployed RRUs and subchannel allocation to minimize energy consumption. Hence, the proposed JEERA algorithm aims to conjointly optimize power allocation, the activated RRUs, subchannel allocation, and user selection allocation to maximize energy efficiency under channel uncertainties. Furthermore, all algorithms are simulated in a similar environment and the simulation outcomes are evaluated over 55 simulations by varying the positions of the IoT devices for the algorithms.

### 5.1. Numerical Results and Discussion

**Figure 2:**
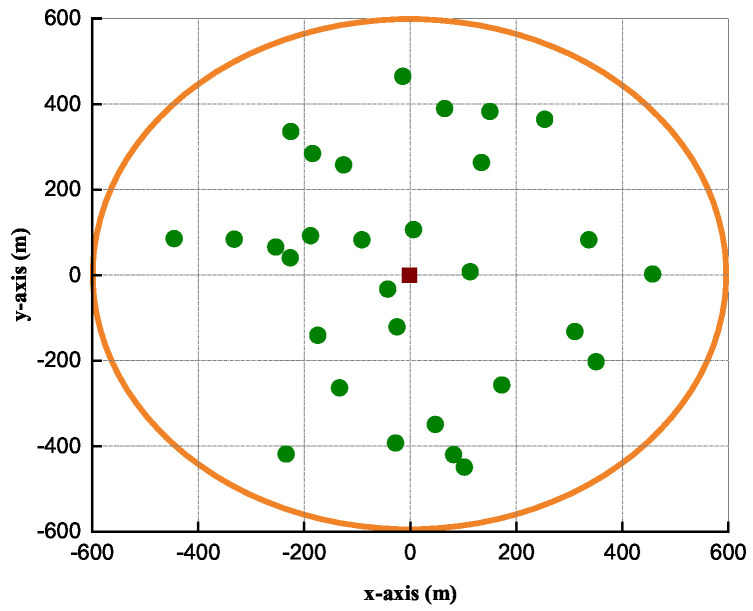
IoT network made up of 30 IoT devices and one RRU.

In Figure 2, 30 IoT users are uniformly distributed within a 600 m circular radius with the connecting RRU at the center. The red square represents the RRU and the small shaded green circles are the IoT devices, respectively.

#### 5.1.1. Effects of Transmission Power on Energy Efficiency

**Figure 3:**
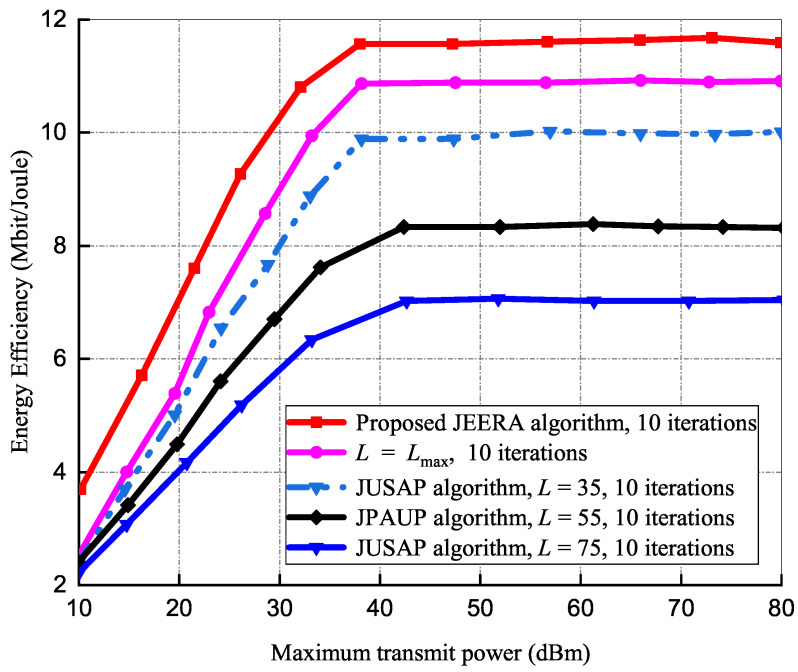
Energy efficiency vs. maximum transmit power.

Figure 3 exemplifies energy efficiency versus maximum transmit power, $P_{\max}$. In this simulation setup, the following parameters were considered: $P_{\max}=80$ dBm, number of iterations at 10, $R_{\min}=2$ bps/Hz, and number of IoT devices at 20, respectively. At $P_{\max}<35$ dBm regime, it is realized that the algorithms have similar energy efficiency performance and they increase linearly as $P_{\max}$ increases. For comparison, JUSAP and JPAUP algorithms have a fixed number of RRUs at L=35, 55, and 75, respectively. At $P_{\max}\geq 40$ dBm regime, the JEERA algorithm activates a lower number of RRUs and therefore its performance reduces as more transmit power is required to satisfy the QoS requirements. When more RRUs are deployed, they increase power consumption, which consequently reduces the system’s energy efficiency functioning. In Figure 3, the proposed algorithm activates fewer RRUs to guarantee QoS requirements with reduced energy consumption. Although *L* performs similarly to the proposed algorithm, it requires extra energy to scale up to handle larger and more complex optimization problems. Thus, the proposed algorithm achieves superior performance in energy efficiency optimization more effectively than conventional algorithms, as the number of connected IoT devices grows exponentially in large-scale IoT environments.

#### 5.1.2. Impact of Transmit Power on Average System Throughput

**Figure 4:**
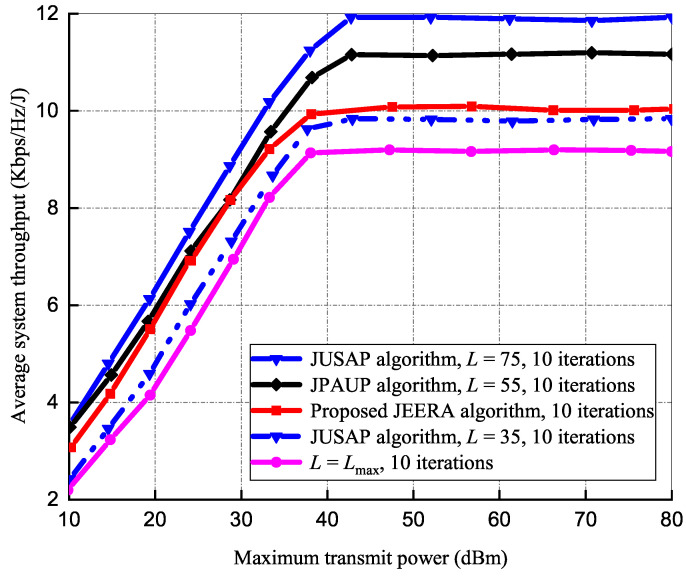
Average throughput of the system vs. maximum transmit power.

Figure 4 presents an average system throughput set against $P_{\max}$ for 20 IoT devices at $R_{\min}$ = 3 bps/Hz and an iteration number of 10, respectively. The average system throughput increases as the $P_{\max}$ increases. At $P_{\max}>40$ dBm regime, it is remarkably noted that the system throughput of the JEERA algorithm reaches a constant. The proposed JEERA algorithm reduces the transmit power to obtain energy efficiency maximization. It is shown that the existing algorithms at L=75 obtain a higher average system throughput with more RRUs due to the high transmit power required. However, the JEERA algorithm obtains better system throughput than the baseline algorithms at $L=L_{\max}$ as it activates a limited number of RRUs. At $P_{\max}<30$ dBm regime, the entire algorithm increases monotonically and shows equal performance gain, as the noise power and the negligence of inter-users interference have consequences on the system’s performance. However, the JEERA algorithm performs better than the JUSAP algorithm at L=35. Thus, the JUSAP algorithm uses inadequate active RRUs and utilizes few energy resources in order to mitigate inter-user interference and to meet the data rate requirement.

#### 5.1.3. Effects of Transmit Power on Total Power Consumption

**Figure 5:**
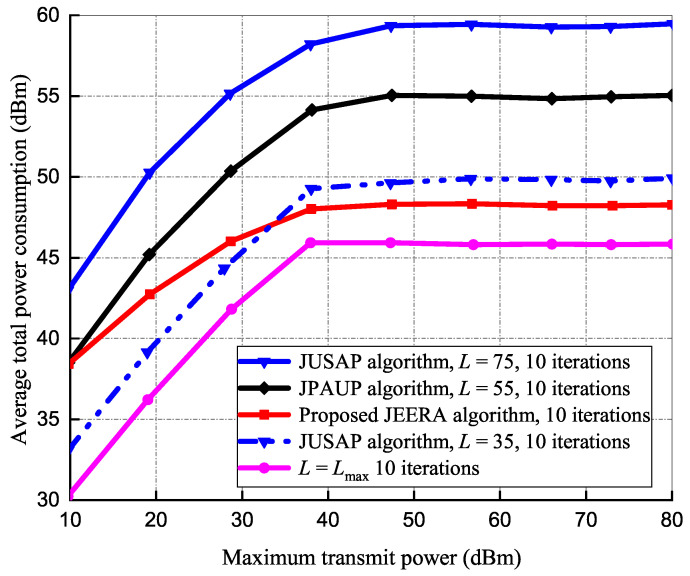
Average power consumption performance.

Figure 5 illustrates the average power consumption set against the $P_{\max}$ in evaluating 20 IoT devices at 10 iterations for all the algorithms’ performances. In $P_{\max}\leq 30$ dBm regime, it can be observed that the JEERA algorithm requires more power at L≤35 than the baseline algorithms because it needs extra activated RRUs to guarantee the data rate provided. The JEERA algorithm steadily increases to constant power consumption as $P_{\max}$ increases. Furthermore, activating extra RRUs nor increasing transmit power substantially benefits the energy efficiency of the system.

#### 5.1.4. Effect of IoT Devices on Energy Efficiency

**Figure 6:**
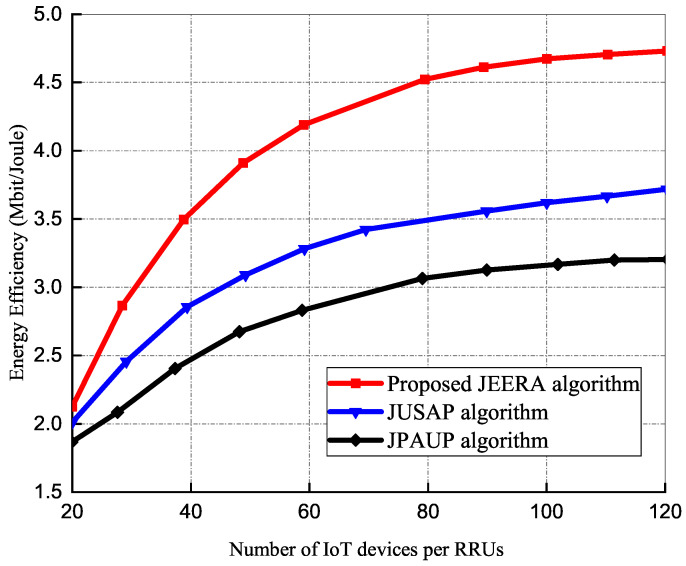
The performance of energy efficiency with number of IoT devices.

In Figure 6, the performance of the energy efficiency with respect to the number of IoT devices is presented. In this simulation, 10 iterations, $P_{\max}$ = 40 dBm, and $R_{\min}$ = 3 bps/Hz and 20 IoT devices are considered. All the algorithms are jointly optimized under the same power constraints and QoS requirements. It is observed in Figure 6 that as the number of IoT devices increases exponentially, there is a gradual increase in energy efficiency performance. The performance gap between the JEERA, JUSAP, and JPAUP algorithms increases as the minimum data rate requirements grow with limited degrees of freedom to utilize the resource allocation effectively. Both JUSAP and JPAUP algorithms utilize large transmit power to maintain the QoS requirement in the presence of imperfect CSI. The JEERA algorithm reaches higher energy efficiency and outperforms the other algorithms due to its high multiuser diversity gain, and in order to have more degrees of freedom when choosing the optimal activated RRUs. When IoT devices are above 30, the JEERA algorithm attains about 33% of the energy efficiency performance, which is higher than the JUSAP algorithm and 37% better than the JPAUP algorithm with an equal power allocation technique.

#### 5.1.5. The Convergence of Proposed Iterative Algorithm

**Figure 7:**
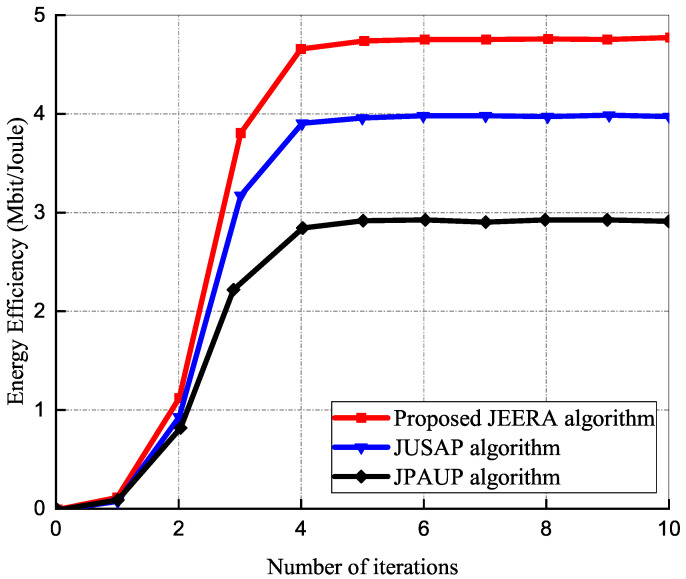
Energy efficiency performance versus number of iterations.

The progress of the JEERA algorithm for different $P_{\max}$ in the RRU and IoT devices is demonstrated in Figure 7. The effects in the figure show over 1500 independent adaptation measures averaged, as each adaptation procedure contains different assignments of multipath fading, path loss, and shadowing. There are 12 uniformly distributed RRUs and all the algorithms converge within 10 iterations. The JEERA algorithm converges slower as compared with the other algorithms, due to its high computational complexity at low SINR regime. However, at high SINR, the performance gain of the energy efficiency reduces for the baseline algorithms. In Figure 7, it is realized that all the algorithms attain saturation and increase monotonically as $P_{\max}$ increases. They achieve optimal energy efficiency and upsurge linearly. Thus, the JEERA algorithm achieves significantly higher energy efficiency than the other algorithms.

**Figure 8:**
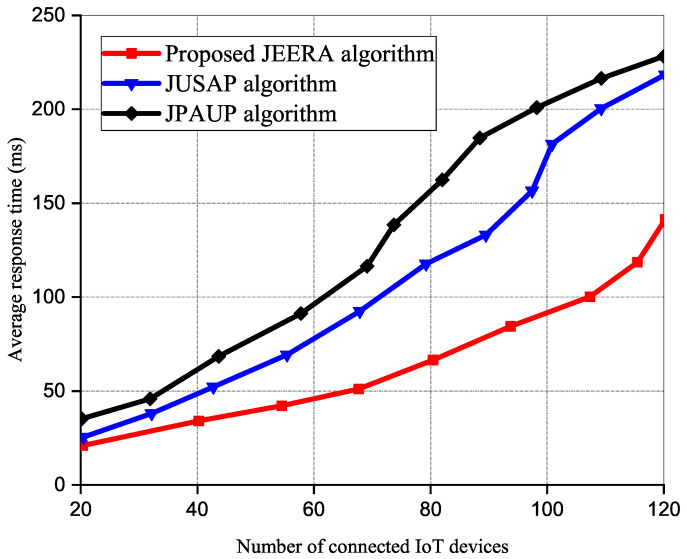
Average response time versus number of IoT devices.

Figure 8 illustrates the average response time versus the connected IoT devices. In our simulation, we gradually increased the number of connected IoT devices and subsequently measured the computational time of all the algorithms in each iteration. The computational time enlarges with several IoT devices connected to the network. However, the proposed JEERA algorithm performs better since it requires less computational time. Hence, it has a low energy consumption rate compared to the existing algorithms. With the increase in number of the IoT devices, the existing algorithms performed similarly.

#### 5.1.6. Impact of SINR Constraints on the Performance of Energy Efficiency

**Figure 9:**
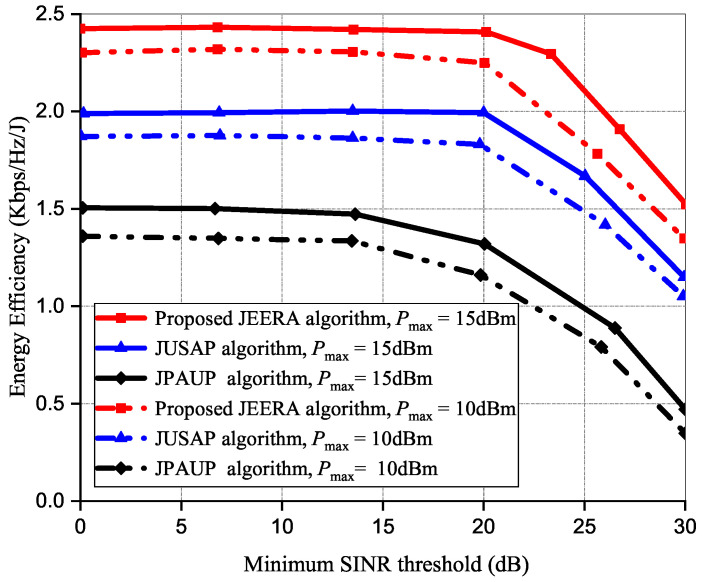
The performance of energy efficiency at different SINR thresholds.

Figure 9 shows the energy efficiency at predefined varying SINR, γ values, and it compares the performances of all the algorithms at a varying $P_{\max}$. Initially, the energy efficiency begins to decline as $P_{\max}$ increases. At γ>12 dB regime, the baseline algorithms have degraded energy efficiency performance in the cascade region. However, the JPAUP algorithm exhibits poorer performance gain as the γ threshold increases. It is observed that $P_{\max}=15$ dB performs better than $P_{\max}=10$ dB, and consequently impacts the energy efficiency performance. Contrarily, at γ<10 dB regime, the JEERA algorithm fully utilizes the available resource allocation at a sufficiently low γ threshold and therefore avoids multi-user interference. The JEERA algorithm achieves better energy efficiency performance than the baseline algorithms.

#### 5.1.7. Effects of Activated RRUs on Transmission Power

**Figure 10:**
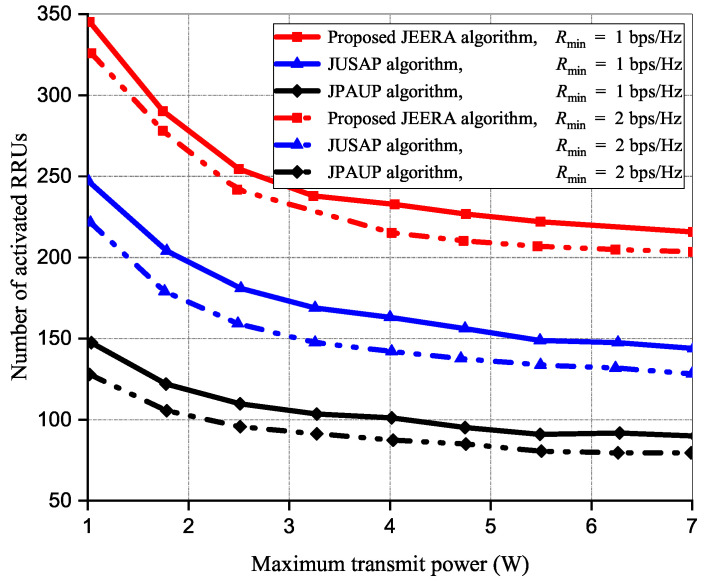
The performance of activated RRUs with maximum transmit power.

Figure 10 investigates the activated RRUs against $P_{\max}$ at varying $R_{\min}$ values. In this simulation, the parameter settings are 15 iterations, 30 IoT devices, $R_{\min}$ = 3 bps/Hz, and 180 RRUs, respectively. It is considered that at large $R_{\min}$ and low $P_{\max}$ W regime, additional RRUs are required to ensure the QoS requirement. The activated RRUs are relatively stable in order to facilitate feasible implementation in IoT networks. However, at small $R_{\min}$ and large $P_{\max}$ regime, the JEERA algorithm introduces a small number of RRUs to optimally improve energy efficiency performance when compared with the baseline algorithms.

## 6. Conclusions

This paper has addressed the resource allocation problem and reformulated it as a joint optimization task by considering the transmission power allocation and the QoS requirement for all the network users under channel uncertainty. As a mixed-integer programming and non-convex problem, it presented no feasible solutions, and due to the computational cost (NP-hard) and strict convexity, the primary problem was then changed to a convex optimization and a parametric tractable form. Furthermore, the main computational task was distributed into various subproblems, which were solved optimally by exploiting the Kuhn–Munkres algorithm and the Lagrangian decomposition approach. Then, a novel low-complexity joint resource algorithm was proposed, which improved energy efficiency performance in dynamic large-scale 6G-IoT ecosystems. The numerical results show that by deploying only a subset of the activated RRUs, the proposed algorithm optimally enhances energy efficiency within practical implementations in IoT networks. Future work will be directed towards exploring the case where each IoT device can be allocated energy resources with multi-objective heuristics strategies.

## Figures and Tables

**Table 1:** List of simulation parameters.

Parameter	Values
Operating frequency	3.8 GHz
Total channel bandwidth	8 MHz
Transmitting antenna gain	12 dB
Path-loss exponent	4
Constant back-off factor	0.3
Noise power per subchannel	−167 dBm
Power amplifier efficiency	0.2
Number of subchannels	32
Power consumption	50 dBm
Minimum data rate	4.2 Mbps
SINR threshold	2.0 dB

## Data Availability

Not applicable.
